# Controlled Transdermal Iontophoresis of Insulin from Water-Soluble Polypyrrole Nanoparticles: An In Vitro Study

**DOI:** 10.3390/ijms222212479

**Published:** 2021-11-19

**Authors:** Kamran Tari, Soroush Khamoushian, Tayyebeh Madrakian, Abbas Afkhami, Marek Jan Łos, Arash Ghoorchian, Mohammad Reza Samarghandi, Saeid Ghavami

**Affiliations:** 1 Department of Environmental Health Engineering, Faculty of Health and Research Center for Health Sciences, Hamadan University of Medical Sciences, Hamadan 6517838636, Iran; kamerantari@yahoo.com; 2 Faculty of Chemistry, Bu-Ali Sina University, Hamedan 6516738695, Iran; soroushkhamoushyian24@gmail.com (S.K.); afkhami@basu.ac.ir (A.A.); arash_ghoorchian@yahoo.com (A.G.); 3 Autophagy Research Center, Shiraz University of Medical Sciences, Shiraz 7135646141, Iran; 4 D-8 International University, Hamedan 65178-38695, Iran; 5 Biotechnology Center, Silesian University of Technology, 8 Krzywousty St., 44-100 Gliwice, Poland; 6 Research Institute of Oncology and Hematology, Cancer Care Manitoba, University of Manitoba, Winnipeg, MB R3E 3P4, Canada; saeid.ghavami@gmail.com

**Keywords:** transdermal iontophoresis, insulin, water soluble polypyrrole, anodal iontophoresis, cathodal iontophoresis

## Abstract

The iontophoresis delivery of insulin (INS) remains a serious challenge due to the low permeability of the drug through the skin. This work aims to investigate the potential of water-soluble polypyrrole nanoparticles (WS-PPyNPs) as a drug donor matrix for controlled transdermal iontophoresis of INS. WS-PPyNPs have been prepared via a simple chemical polymerization in the presence of sodium dodecyl sulfate (SDS) as both dopant and the stabilizing agent. The synthesis of the soluble polymer was characterized using field emission scanning electron microscopy (FESEM), dynamic light scattering (DLS), fluorescence spectroscopy, and Fourier transform infrared (FT–IR) spectroscopy. The loading mechanism of INS onto the WS-PPyNPs is based on the fact that the drug molecules can be replaced with doped dodecyl sulfate. A two-compartment Franz-type diffusion cell was employed to study the effect of current density, formulation pH, INS concentration, and sodium chloride concentration on anodal iontophoresis (AIP) and cathodal iontophoresis (CIP) of INS across the rat skin. Both AIP and CIP delivery of INS using WS-PPyNPs were significantly increased compared to passive delivery. Furthermore, while the AIP experiment (60 min at 0.13 mA cm^–2^) show low cumulative drug permeation for INS (about 20.48 µg cm^−2^); the CIP stimulation exhibited a cumulative drug permeation of 68.29 µg cm^−2^. This improvement is due to the separation of positively charged WS-PPyNPs and negatively charged INS that has occurred in the presence of cathodal stimulation. The obtained results confirm the potential applicability of WS-PPyNPs as an effective approach in the development of controlled transdermal iontophoresis of INS.

## 1. Introduction

The increase of obesity incidence in virtually all countries around the world drives the rise in diabetes (mostly type-II diabetes) occurrence [1,2]. It was estimated by the International Diabetes Federation (IDF) that diabetes was responsible for 4.2 million deaths around the world in 2019, which is equivalent to one death every seven seconds [3]. Currently, controlling blood glucose concentration through the subcutaneous injection of insulin (INS) is the most effective treatment of diabetes [4]. Unfortunately, the treatment with self-injections can be associated with inflammation, pain, local tissue necrosis, and the risks of microbial contamination [5]. Hence, a wide range of INS delivery methods have been employed as alternatives for daily INS therapy, including oral, pulmonary, nasal, and transdermal approaches [1,6,7,8]. The transdermal drug delivery methods have remarkable advantages compared with others, such as increased patient acceptance, simplicity, and lower potential for injection site-associated infections [9,10].

As one of the most popular transdermal drug delivery methods, iontophoresis (IP) has advantages of practicality, simplicity, low cost, and portability [11,12]. IP is a non-invasive technology that employs a low electric current (≤0.5 mA cm^−2^) to drive a drug into and across the skin using two electrodes. Basically, the electrodes are positioned over two different regions of the skin. The penetration of the drug through the skin is dependent on the polarity of the electrodes and the physicochemical properties of the formulation [13]. The development of IP delivery of INS has been limited due to the low permeability of the drug through the skin that hinders its bioavailability [14,15]. Many attempts have been concentrated on developing IP delivery systems to improve the permeation rates of INS crossing the tissue barriers. For instance, Chen et al. employ microneedle-induced skin microchannels to enhance the permeation rate of INS from INS-loaded nanovesicles [16]. The combination of solid microneedle arrays with an IP approach leads to provide a synergistic enhancement of INS permeation. Many other studies tried to address this question using a wide range of chemical permeation enhancers [17,18,19]. As reported, attempts have been made to utilize nanoparticles to increase the performance of controlled drug delivery [20,21,22].

An interesting class of macromolecules, known as water-soluble conjugated polymers, has been introduced in recent years [23], which has opened up new research avenues in biomedical applications [24,25]. They have attracted tremendous attention due to their applications in imaging, drug- and gene delivery, and anticancer therapy. One prime candidate for designing water-soluble conjugated polymers is the use of surfactants as stabilizing agents [26,27]. The incorporation of surfactants can increase interactions between the polymer backbone and water molecules, leading to improved solubility of these macromolecules. Polypyrrole (PPy) is a versatile member of conjugated polymers, which has exhibited extraordinary properties, including biocompatibility, good electrical conductivity, ease of synthesis, and environmental stability [28]. Several efficient methods for preparing a series of water-soluble PPy nanoparticles (WS-PPyNPs) have been reported [27,29,30,31].

**Figure 1:**
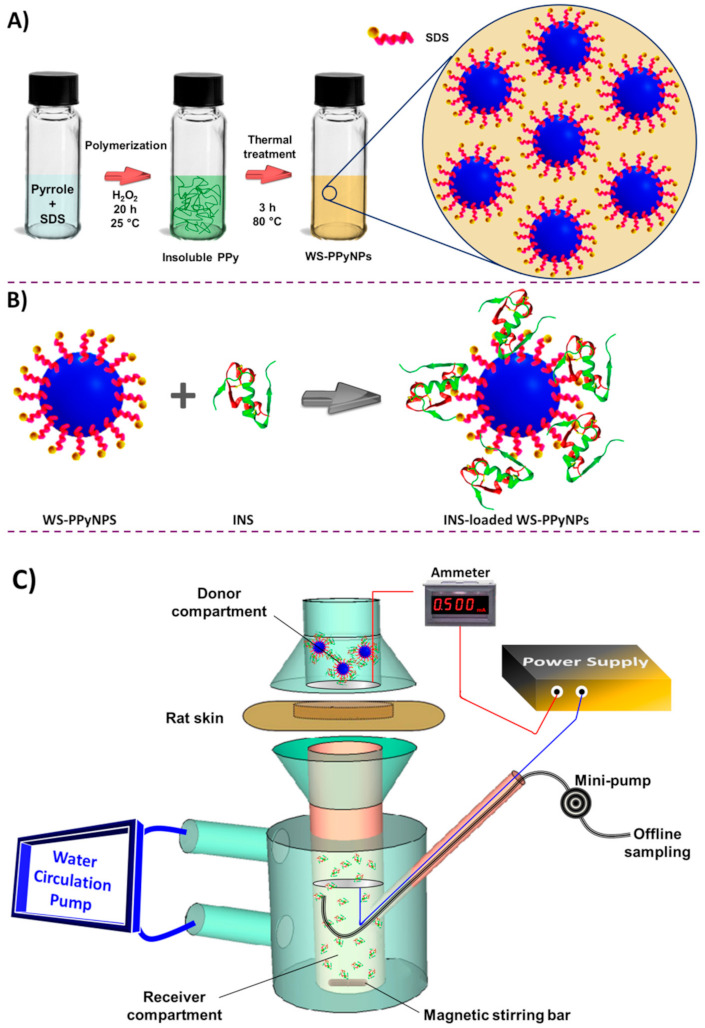
Schematic overview of main experimental procedures. (**A**) Schematic overview of step-by-step synthesis of WS-PPyNPs. (**B**) The preparation of INS-loaded WS-PPyNPs. (**C**) Experimental set up of in vitro controlled transdermal iontophoresis of INS-loaded WS-PPyNPs.

Given the rationale described above, the objective of this work is to evaluate the potential of a water-soluble polymer, as an efficient drug donor matrix for iontophoretic delivery of INS. On this account, we studied the effect of different experimental parameters on the iontophoretic transport of the drug across the rat skin using WS-PPyNPs, in in vitro experiments (Figure 1). At first, WS-PPyNPs were prepared using sodium dodecyl sulfate (SDS) as the stabilizing agent. Afterward, INS was loaded onto the nanoparticles to obtain INS-loaded WS-PPyNPs. The employment of WS-PPyNPs associated with the IP approach can increase the transdermal transport rate of INS. To assess the delivery of the drug, the iontophoretic profiles of INS from INS-loaded WS-PPyNPs during anodal and cathodal stimulation were examined, which suggests that this method can be applicable for maintaining the blood glucose concentration.

## 2. Results and Discussion

### 2.1. Characterization of the Obtained WS-PPyNPs

**Figure 2:**
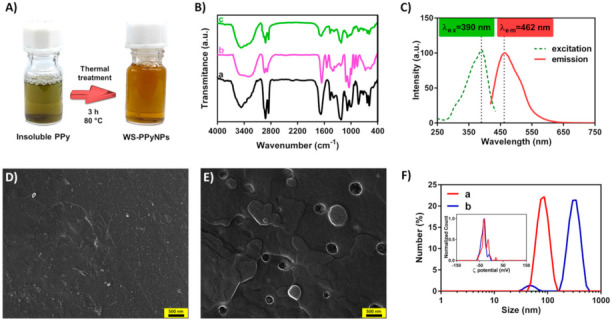
Assessment of the conversion of PPy to WS-PPyNPs. (**A**) The procedure for the conversion of insoluble PPy to WS-PPyNPs. (**B**) FT-IR spectra of (a) WS-PPyNPs, (b) INS, and (c) INS-loaded WS-PPyNPs. (**C**) Fluorescence excitation and emission spectra of 200-times diluted WS-PPyNPs. FESEM image of (**D**) WS-PPyNPs, and (**E**) INS-loaded WS-PPyNPs. (**F**) Size distribution and ζ potential (the inset) profiles of (a) WS-PPyNPs and (b) INS-loaded WS-PPyNPs obtained using DLS. Scale bar = 500 nm.

The WS-PPyNPs were prepared in the aqueous solution as described in the experimental section. At first, pyrrole was polymerized using a chemical polymerization in the presence of dodecyl sulfate (DS), as a doping agent. To obtain a water-soluble polymer and to facilitate the incorporation of DS ions into the PPy backbone, the polymer suspension was thermally treated. During the heat treatment step, the insoluble aggregates slowly disappeared, and consequently, a homogeneous solution was achieved, as shown in Figure 2A. The incorporation of DS (as an electrostatic stabilizer and dopant) into the polymer backbone leads to increasing the water solubility of PPy. It should be pointed out that the biocompatibility of PPy has been reported previously [32,33].

The FT-IR spectra of WS-PPyNPs, INS, and INS-loaded WS-PPyNPs samples are depicted in Figure 2B. As could be seen, the FT-IR spectrum of WS-PPyNPs displays characteristic peaks at 1465 cm^−1^, 1680 cm^−1^, 1074 cm^−1^, 1383 cm^−1^, and a broad peak at 3400 cm^−1^ which is ascribed to the symmetric and asymmetric ring-stretching vibrations [34], C–H and C–N in-plane deformation vibrations [35], and N–H and C–H stretching modes [36], respectively. Furthermore, an absorption peak appears at 1232 cm^−1^, which is related to the S=O stretching vibration of DS anion [37], revealing that DS anions are incorporated into the PPy backbone during the polymerization process. It is concluded that the WS-PPyNPs are successfully prepared. As can be observed from Figure 2B (curve b), the FT-IR spectrum of INS shows a characteristic peak at 1651 cm^−1^ (C=O stretching of amide-I), which is related to the α-helical structure, revealing that INS exists in its native form [38]. A peak at 1539 cm^−1^ is ascribed to the amide-II corresponding to the C–N stretching and N–H bending modes [39,40]. Furthermore, a very broad band is clearly observed at around 3300 cm^−1^ and attributed to the N–H stretching vibrations [41]. From Figure 2B (curve c), the characteristic peaks of INS are observed in INS-loaded WS-PPyNPs. Hence, the INS is successfully preserved in the structure of INS-loaded WS-PPyNPs. The synthesis of WS-PPyNPs was also studied using fluorescence spectroscopy. Figure 2C reveals the fluorescence excitation and emission spectra of 200-times diluted WS-PPyNPs sample. The fluorescence excitation and emission wavelengths obtained for the as-prepared nanoparticle were 390 and 462 nm, respectively, in agreement with published results [31]. The FESEM technique was used to investigate the morphology of WS-PPyNPs, and INS-loaded WS-PPyNPs samples. An island-like structure of the PPy was observed from the FESEM image of the as-prepared WS-PPyNPs (Figure 2D). As a result of the loading of INS on the WS-PPyNPs, the surface morphology obviously converted to a smooth surface, as indicated in Figure 2E. It can be seen that the observed morphology of INS loaded-WS-PPyNPs was different from that of WS-PPyNPs.

Finally, the particle size of WS-PPyNPs was measured using DLS technique. It is known that DLS is a common technique to measure the sizes of nanoparticles [42], and it has been successfully utilized to confirm the drug loading [43,44]. From Figure 2F, during the drug loading, the average size of the WS-PPyNPs changed from 108.6 nm to 423.5 nm, indicating the successful loading of INS onto the WS-PPyNPs. Further, the inset of Figure 2F shows the ζ potential value at around −38 mV for WS-PPyNPs and INS loaded-WS-PPyNPs, respectively, revealing that both nanoparticles carried with negative charge. 

### 2.2. Stability of INS

The drug was stable in the presence of a constant current (0.13 mA cm^−1^ for 48 h) with a recovery of 93.1%. This confirmed that the stability of INS is sufficiently high to study transdermal delivery. Further studies are planned to assess long-term drug stability.

### 2.3. The Loading Mechanism 

**Figure 3:**
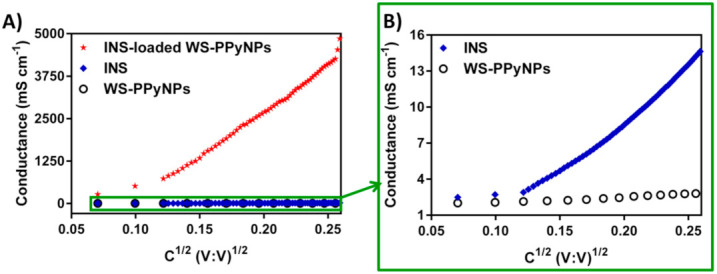
Conductivity assessment of INS-loaded WS-PPyNPs. (**A**) Plots of conductivity, versus square root concentration in water at 25 °C, WS-PPyNPs (blank circle), INS (blue diamond), and INS-loaded WS-PPyNPs (red asterisk). (**B**) High-resolution conductometric curves of WS-PPyNPs and INS.

To assess the loading mechanism of INS onto the surface of WS-PPyNPs, a conductometric investigation was employed. Figure 3A indicates the conductance vs. the square root concentration of pure INS, WS-PPyNPs, and INS-loaded WS-PPyNPs samples in a 0.1 mol L^−1^ PBS (pH = 7.4). To better illustrate the conductance behavior of pure INS and WS-PPyNPs, Figure 3B shows the same conductometric data of Figure 3A on an enlarged scale. As expected, the PPy solution has no conductivity, a result that is in agreement with a previously published work [45]. In an aqueous solution, the hydrophobically attached DS ions undergo dissociation to produce free ions, leading to an increase in the conductivity of the studied solution. Contrarily, DS ions that are incorporated as anionic dopants are electrostatically bonded to positively charged sites of PPyNPs, and consequently, dissolving them in an aqueous solution will not increase the conductivity. It could be concluded that all of the presented DS ions in the PPy backbone act as the anionic dopant. The obtained results demonstrate that the pure INS has almost no conductivity.

Interestingly, INS-loaded WS-PPyNPs show significant changes in conductivity. In the case of INS-loaded WS-PPyNPs, INS can be replaced with doped DS anions, generating free ions and increasing the conductivity. For better visualization purposes, Figure 1B illustrates the proposed mechanism for the loading of INS on the surface of WS-PPyNPs. 

### 2.4. Optimization of Experimental Conditions 

#### 2.4.1. Effect of Current Density

**Figure 4:**
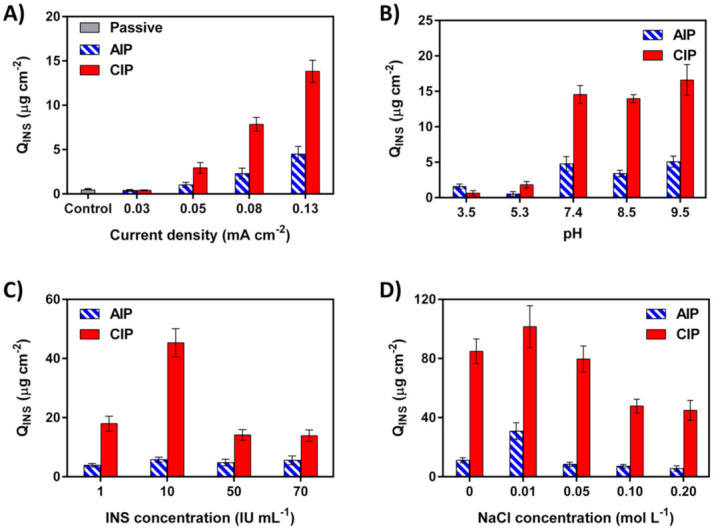
The effect of (**A**) current density, (**B**) formulation pH, (**C**) INS concentration, and (**D**) sodium chloride concentration on the AIP and CIP of INS from INS-loaded WS-PPyNPs.

The drug transport in the skin is preferentially affected by the current density. Thus, prior to study the transdermal transfer of INS, we had to optimize the applied current density during AIP and CIP experiments. The iontophoretic transdermal transfer of INS was carefully assessed in the different current densities employing 50 IU mL^−1^ INS (Figure 4A). As expected, the passive permeation of the drug was very low compared to AIP and CIP. Hence, the electrical stimulation (ion transport) enhanced the movement of drugs across the skin. 

The results show that CIP of INS at 0.03, 0.05, 0.08, and 0.13 mA cm^−2^ resulted in a cumulative transdermal transfer of 0.41, 2.93, 7.85, and 13.83 µg cm^−2^, respectively. Similarly, in AIP stimulation, the cumulative iontophoretic transdermal transfer increased as the current density was increased from 0.03 to 0.13 mA cm^−2^. Therefore, 0.13 mA cm^−2^ was chosen as the optimum current density for both AIP and CIP, because they induce the highest transdermal transfer of INS. Hence the current density could be employed to control drug delivery into the skin. 

The amount of INS transdermally transferred during CIP stimulation was significantly enhanced compared to that of AIP stimulation. This could be explained by the repulsion of negatively charged INS at the cathode (the negative pole). As expected, an anionic drug (such as INS) transports from the cathode reservoir to the anode [46]. However, it should be noted that the positive charge of PPy as a carrier plays a pivotal role in the explanation of this observation. In other words, the separation of positively charged WS-PPyNPs and negatively charged INS has occurred in the presence of cathodal stimulation. This fact was observed as well by Hosseini-Nassab et al. [47] and Rossi et al. [48].

#### 2.4.2. Effect of pH of the Formulation

In the iontophoresis, the formulation pH governs the stability of the drug and may affect the transport across the skin. Thus, to obtain the best delivery performance, the effect of formulation pH in AIP and CIP delivery of INS using WS-PPyNPs was evaluated (Figure 4B). The effect of formulation pH was investigated in the pH-range 3.5−9.5. Minimum permeations for INS were achieved at pH ≤ 5.3 during AIP and CIP experiments. This likely could be explained by the isoelectric point (pI) of INS at pH 5.3 [49]. That is why a monomeric INS analogs showed higher iontophoretic mobility in pH > 7.4 than native INS [50]. At pH values > 5.3 the charge of INS became negative (See the inset of Figure 2F), resulting in the repulsion between the charged drug and cathode, and the transdermal transfer is enhanced. 

Biological activity of the majority of drugs is influenced by pH. It has previously been reported that the lower amount of transdermally transferred INS during AIP and CIP at pH 5.3 is due to the change in INS conformation and the drug degradation [49,51]. Hence, the biological activity (hypoglycaemic activity) of the drug is very low at this pH. The CIP of INS at pH above pI is preferred because of to the excellent stability of the drug [49]. Consequently, pH 7.4 that produced the maximum drug permeation was selected as the optimum pH for AIP and CIP experiments. The pH 7.4 also offers best biocompatibility of the tested iontophoretic transdermal transfer protocol.

#### 2.4.3. Effect of INS Concentration

The transdermal delivery experiments were performed while employing different concentrations of INS (from 1.0 to 70 IU mL^−1^) to understand the effects of the drug concentration on AIP and CIP delivery of INS. When 10 IU mL^−1^, 50 IU mL^−1^, and 70 IU mL^−1^ of the drug were entrapped into the polymer backbone, the entrapment efficiencies were 92.41%, 83.63%, and 62.46%, respectively (Table S1). In both stimulations, the results illustrate that the maximum drug permeation was obtained in the presence of 10 IU mL^−1^ INS, while higher concentrations lead to a considerably lower permeation, as depicted in Figure 4C. This is likely caused by the aggregation of INS to form dimers and hexamers, depending on the concentration and pH of the solution. INS aggregation is considered an undesired process, because it increases the macromolecular size, and decreases the bioactivity of the drug [52]. Other researchers reported similar observations in the iontophoretic delivery of INS [52,53]. Consequently, 10 IU mL^−1^ was selected as an optimal value of the INS concentration to provide the maximum drug permeation.

#### 2.4.4. Effect of Sodium Chloride Concentration

The transport number of an ion, which represents the portion of the total current carried, is governed by its mobility and concentration. Compared to other ions present in the solution, drug ions usually have a low transport number due to their low mobility and concentrations [54]. Consistent with this fact, INS, as a large peptide, has a low transport number [55]. During iontophoresis, other ions compete with the drug for transport across a membrane (transdermal) depending on their transport numbers [56]. Hence, the concentration of NaCl in the donor compartment plays a crucial role in the transdermal transport of INS. Results presented in Figure 4D show the efficacy of INS transdermal transport during AIP as well as CIP in the presence of different concentrations of NaCl. Interestingly, in both types of experiments, the cumulative amount of drug transdermally transferred within 20 min increased with the concentration of salt and reached a maximum at 0.01 mol L^−1^, and followed by a decrease. The observed behavior can be attributed to many causes. (i) The iontohydrokinesis, where ions carry water molecules across the different types of pore in the membrane. The ion-induced convective flow in a charged membrane is significantly increased in the presence of high concentrations of NaCl [57,58]. Hence, at higher concentrations of NaCl, the saturation of pores in the skin causes an increase in the transport of ions and water molecules into the pores and a decrease in the amount of INS. It should be noted that the ion-induced convective flow in the transport of a large drug (such as INS) is of more importance compared to small polar drugs [59]. (ii) Pillai et al. reported that high concentrations of NaCl lead to more tendency of INS to deposit in the skin and subsequently to decrease INS permeated [52]. They found that the electro-osmotic flow significantly decreased at higher concentrations of NaCl. (iii) The aggregation of INS surges by increasing the concentration of NaCl, leading to a decrease in the permeated INS [60]. Hence, 0.01 mol L^−1^ was selected as the optimum salt concentration in AIP and CIP experiments.

### 2.5. INS Transdermal Delivery

**Figure 5:**
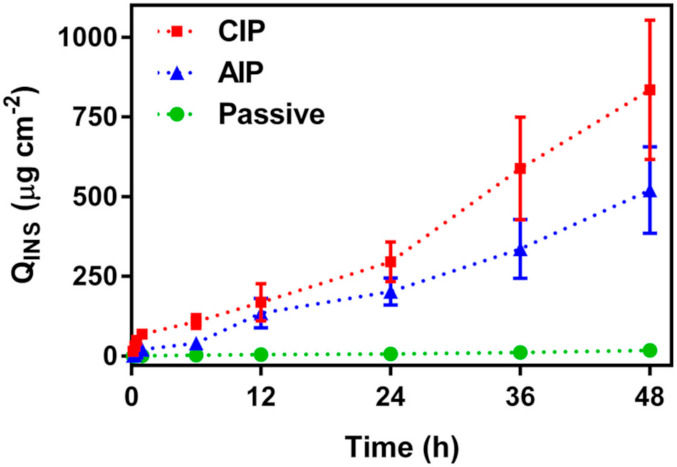
Permeation curves of INS-loaded WS-PPyNPs after 48 h under passive, AIP and CIP conditions. Red squares and blue triangle denote INS permeation upon the application of CIP and AIP, respectively, while green circles represent INS permeation under passive condition.

The transdermal delivery of INS across the rat skin was examined for 48 h under passive, as well as iontophoretic conditions. The AIP and CIP experiments were carried out applying a constant current density of 0.13 mA cm^−2^. The Q values of permeated INS were recorded at different times, and the obtained results are presented in Figure 5. 

To achieve the pseudo-steady-state flux, and lag time, the permeation curves (Q vs. time) from 6 to 48 h were used. The cumulative amount of permeated INS in 48 h (Q48h), flux, lag time, P, and EF are listed in Table 1. 

It should be emphasized that the application of a low current density (≤0.5 mA cm^−2^) is the main advantage of the iontophoresis technique. The current density of 0.13 mA cm^−2^ was applied to samples in all iontophoretic experiments in order to enhance the permeation rate of INS across the rat skin. As expected, the applied current density of 0.13 mA cm^−2^ exhibited a significant increase in cumulative drug permeation under iontophoretic conditions, compared to passive permeation, by enhancing Q48h. The INS-loaded WS-PPyNPs under each iontophoretic stimulations permeated more than 750 µg cm^−2^ INS on average for 48 h. After 48 h, 834 µg cm^−2^ was permeated across the skin to which the cathodal current was used compared to only approximately 48 µg cm^−2^ in the passive experiment. Notably, prescribed concentration of INS is ~18–24 IU (~624–833 µg) [61]. Moreover, thanks to the ability of IP technique to utilize working electrode with different sizes, the proposed delivery technique can provide the required INS level for diabetics. The flux values of passive, AIP, CIP were obtained to be 0.36, 9.59, and 15.67 µg cm^−2^ h^−1^, respectively, indicating that electrical stimulation affects the INS delivery. Moreover, the AIP and CIP experiments indicated lag times equal to approximately 100 min. The relatively high lag time values resulted from the fact that INS accumulated in the skin and consequently slowly released [49,55]. This is due to the high skin affinity of INS.

The P_app_ of INS across the rat skin was found to be 7.68 × 10^−6^ cm s^−1^ and 1.25 × 10^−5^ cm s^−1^ under anodal and cathodal stimulations, respectively, while that was 2.88 × 10^−7^ cm s^−1^ in the passive permeation (Table 1). The values obtained for P_app_ were comparable with the values reported in the literature on iontophoresis delivery of INS [62]. In the mentioned work, the Q values of permeated INS after 5 h is significantly lower than that of the presented work. This is due to the presence of WS-PPyNPs as an efficient drug donor matrix for iontophoretic delivery of INS. Furthermore, our work provides a more comprehensive in vitro IP iontophoretic delivery of INS that studies the effective experimental parameters. The results demonstrate that iontophoretic stimulation is effective in increasing drug permeation into the skin barrier.

**Figure 6:**
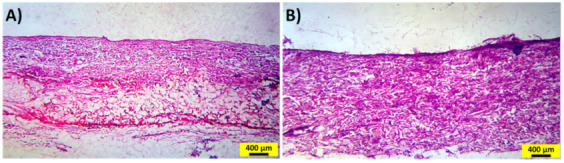
Representative H&E staining assay of rat skin after the IP experiment, except for (**A**) the control, (**B**) applying the current density of 0.13 mA cm^−2^ for 48 h. Scale bar = 400 μm.

To evaluate the possible negative effect of iontophoretic delivery on the rat skin, Hematoxylin-eosin (H&E) staining assay was used, as shown in Figure 6. In the histological images of H&E-stained skin tissues, no significant increase in inflammatory cells was observed in the sample treated by applying the current density of 0.13 mA cm^−2^ for 48 h. A similar observation was reported for transdermal delivery of an antibody via IP on the dorsal skin of rats [63]. Hence, the proposed method is a non-invasive and biocompatible technology for transdermal drug delivery.

## 3. Experimental Procedures

### 3.1. Materials

Pyrrole, hematoxylin, and eosin solutions were obtained from Sigma Aldrich (Steinheim, Germany). Hydrogen peroxide (H_2_O_2_, 30% *w*/*w*), SDS, disodium hydrogen phosphate (Na_2_HPO_4_), and sodium dihydrogen phosphate (NaH_2_PO_4_) were procured from Merck Company (Darmstadt, Germany). Human regular INS was purchased from Exir Pharmaceutical Co., (Tehran, Iran). All other materials used were analytical reagents as purchased without any further purification. A 0.1 mol L^−1^ phosphate-buffered saline (PBS, pH = 7.4) was obtained by mixing an appropriate amount of Na_2_HPO_4_, NaH_2_PO_4_, and NaCl. The pH of PBS was adjusted by NaOH and H_3_PO_4_ solution. 

### 3.2. Synthesis of WS-PPyNPs

The WS-PPyNPs were prepared by a chemical polymerization method [31,47], with some modifications. First, 2.9 mmol of pyrrole and 2.9 mmol of SDS were added to 35 mL of deionized water and then ultrasonicated for 10 min. Subsequently, 30.9 mmol of H_2_O_2_, as the oxidizing agent, was carefully added to the above solution drop by drop and kept under vigorous stirring at room temperature. By the end of 20 h of stirring, an insoluble PPy with dark green color was observed, revealing the polymerization of PPy. Then, to achieve the WS-PPyNPs, simple thermal treatment was used at 80 °C for 3 h through continuous stirring, and subsequently cooled slowly to room temperature. The apparent color of the polymer solution turns from green to brown at the end of the reaction. The as-synthesized polymer solution was filtered through a 0.45 μm Whatman^®^ fitter paper to separate any sediment. An illustration of the synthesis pathway of WS-PPyNPs is presented in Figure 1A. 

### 3.3. INS Loading Experiments

A 500 μL of different concentrations of INS solution was mixed with a 500 μL of WS-PPyNPs solution by magnetic stirring for 30 min, as illustrated in Figure 1B. To avoid degradation of the drug, all of loading experiments were performed at room temperature. The entrapment efficiency (EE) of the drug in WS-PPyNPs was obtained by the determination of the amounts of free INS. The EE% was calculated from Equation (1):

(1) $$\mathrm{EE}\%=\frac{\mathrm{added}\;\mathrm{INS}-\mathrm{free}\;\mathrm{INS}}{\mathrm{added}\;\mathrm{INS}}\times 100$$

The concentration of free INS in the INS-loaded WS-PPyNPs sample was calculated based on the calibration curve established by plotting the fluorescence intensity of INS monitored at 301.5 nm (at an excitation wavelength of 260 nm) vs. their known concentrations. The EE% values were calculated using fluorescence spectroscopy, and presented in Table S1. 

### 3.4. Characterizations of the Obtained and INS-Loaded Matrix

Fourier transform infrared (FT–IR) spectroscopic studies of insoluble PPy, WS-PPyNPs, and INS-loaded WS-PPyNPs were carried out on a PerkinElmer (Model Spectrum GX) infrared spectrometer. The morphological properties and size measurement of WS-PPyNPs and INS-loaded WS-PPyNPs were assessed using a field emission scanning electron microscope (FESEM). To form a thin film, a 10 μL of 100 IU mL^−1^ INS was mixed with a 10 μL of WS-PPyNPs solution and then dropped onto the surface of an aluminum substrate. Additionally, a 20 μL of WS-PPyNPs solution without drug loading was coated in the same way. Prior to imaging, the samples were dried in a vacuum desiccator. FESEM images were collected by a TESCAN MIRA3 LMU microscope (Czech Republic). Particle size and ζ potential measurements were evaluated by dynamic light scattering (DLS) using a Zetasizer NanoZS (Malvern Instruments Ltd., Worcestershire, UK) instrument at 25 °C with an incident He-Ne laser (633 nm). For DLS measurements, 5 µL of the WS-PPyNPs, and INS-loaded WS-PPyNPs were diluted with 950 µL of deionized water. Fluorescence measurement was conducted by a PerkinElmer fluorescence spectrometer using emission and excitation band-passes of 10 and 10 nm and a scan rate of 200 nm min^−1^. The conductance measurements were carried out at room temperature using a Metrohm conductometer (Model 712, Swiss) equipped with platinized electrodes (cell constant = 0.97 cm^−1^).

### 3.5. Skin Preparation

In IP experiments, to represent human skin, various animal skins (rodent, pig, rabbit and snake) are used for two reasons; (i) since electroosmosis is one of the main permeation mechanisms, the used skin should be cation-selective, and (ii) the hair follicle density of the animal skin should be close to that of human skin [64]. With respect to these two requirements, pig skin has usually been employed as an in vitro model for IP. However, in transdermal IP, the electroosmotic flow across pig skin has been reported to be much higher than that of human skin. Fortunately, rat skin satisfies the above-mentioned requirements [65,66]. Moreover, rat skin is low-cost, easily accessible, and very comparable to human skin [66]. Therefore, in this work, rat skin was chosen as the in vitro model.

All animal experimentation protocols were conducted in accordance with the guidelines approved by the National Institute for Medical Research Development (NIMAD) ethical committee (IR.NIMAD.REC.1398.380). Healthy adult male Wistar albino rats (average body weight: 180 ± 10 g) were obtained from Hamedan University of Medical Sciences (Hamadan, Iran) and were 8 weeks old at the start of each experiment. The animals were first anesthetized and then sacrificed using urethane injection, and the hair of the dorsal area was removed using a depilatory and an animal hair clipper. Afterward, the subcutaneous fat was carefully removed using a surgical scalpel. Subsequently, the skin samples were completely washed with deionized water and stored in a refrigerator at −26 °C for a maximum period of 10 days.

### 3.6. In Vitro Permeation Experiments

The skin sample was clamped in a two-compartment Franz-type diffusion cell (area 3.80 cm^2^). The receiver compartment was filled with 8 mL of PBS (pH 7.4), followed by the equilibration for 30 min at 37 °C. The donor compartment (formulation compartment) was then filled with 1 mL of different INS-loaded WS-PPyNPs formulations (pH 7.4). The side-arm, including offline sampling and a stainless steel electrode (2 cm diameter), was connected to the receiver compartment. Constant current densities were applied through two stainless steel electrodes connected to a commercially available power supply (TSi Co., model TPS-3010U). The applied current density was carefully controlled using an ammeter (precision ± 0.001 mA). A schematic illustration of the in vitro permeation setup is shown in Figure 1C. In the anodal iontophoresis (AIP), the anode was positioned in the donor compartment and the cathode in the receiver compartment. During cathodal iontophoresis (CIP), the electrode polarity was reversed. Passive permeation experiments were performed as a control without applying any current, and response behaviors were compared with AIP and CIP, respectively. After each in vitro experiment, 300 µL of the receptor compartment was sampled at different time-points. Then, INS concentration was immediately measured by integration of absorption spectra in the region of 257–317 nm using an Agilent diode array spectrophotometer (Agilent 8453, Milford, MA, USA). The absorbance spectra of PBS (pH 7.4) in the presence of various concentrations of INS were illustrated in Figure S1. Good linearity was obtained from 0.09 to 5.45 IU mL^−1^ with a correlation coefficient of 0.9995. The limit of detection was achieved as 0.03 IU mL^−1^, which is satisfactory for the quantification of permeated INS in the receiver compartment. Further, to validate the concentration of permeated INS, the fluorescence spectroscopy was used. The fluorescence spectra were recorded over the wavelength range of 260−760 nm at an excitation wavelength of 260 nm at room temperature. The fluorescence spectra and the corresponding fluorescence intensity at 301.5 nm in the presence of various concentration of INS were recorded and shown in Figure S2. All experiments were carried out at least thrice.

The cumulative amount of permeated INS per cm^2^ of rat skin (Q, µg cm^−2^) was plotted as a function of time, and the permeation parameters were obtained [67]. The flux was obtained as the slope of the permeation curve at pseudo-steady-state. The lag time was calculated by simplistic extrapolation of the linear portion of the permeation curve. The apparent permeability coefficient (P_app_, cm s^−1^) at pseudo-steady-state is defined by the following equation:

(2) $$P_{\mathrm{app}}=\frac{f}{C_{\mathrm{INS},D}\;}\;$$

where f is the flux (µg cm^−2^ s^−1^) and C_INS,D_ (µg cm^−3^) is the INS concentration in the donor compartment. Further, the enhancement factor (EF) was obtained from Equation (3):

(3) $$\mathrm{EF}=\frac{P_{\mathrm{app},\;\mathrm{iontophoresis}}}{P_{\mathrm{app},\mathrm{passive}}\;}\;$$

### 3.7. Effect of Experimental Conditions on IP

The one-factor-at-a-time method was employed to optimize the effective parameters on IP and tests factors individually. Previous studies about optimization in IP are based on one-factor-at-a-time method [49]. To study all experimental parameters on IP, 20 min was selected as the time of IP as this duration time is greater than the reported retention time of INS (9 min) [49,52].

First, the effect of current density on the AIP and CIP was examined using an INS solution (50 IU mL^−1^, pH 7.4), by applying different current densities (0.03, 0.05, 0.08, and 0.13 mA cm^−2^*)* for 20 min.

The effect of formulation pH on the AIP and CIP stimulations of the drug was investigated by comparing INS permeated for 20 min from different donor solutions containing 50 IU mL^−1^ INS at pH 3.5, 5.3, 7.4, 8.5, and 9.5.

The concentration of INS in the donor solution was varied from 1 to 70 IU mL^−1^ in 0.2 mol L^−1^ NaCl solution. The current density was fixed at 0.13 mA cm^−2^ in both AIP and CIP experiments.

To evaluate the effect of sodium chloride on the delivery, the salt concentration in the donor compartment was varied from 0.01 to 0.20 mol L^−1^, while the concentration of INS was constant (10 IU mL^−1^).

### 3.8. Stability of INS in the Presence of Electrical Current

The stability of INS in the presence of electric current was studied via applying a constant current density of 0.13 mA cm^−1^ during 48 h to a 0.1 mol L^−1^ PBS (pH = 7.4) containing 10 IU mL^−1^ INS. Then, the concentration of INS was quantified by absorption spectroscopy.

## 4. Conclusions

The present work introduced WS-PPyNPs as an efficient drug donor matrix for transdermal delivery of INS using a low current density (0.13 mA cm^–2^). The results demonstrate that INS was successfully loaded onto the surface of WS-PPyNPs. In vitro experiments were performed using INS-loaded WS-PPyNPs to transdermally transport the drug across the rat skin. The effects of experimental parameters, including current density, formulation pH, INS concentration, and sodium chloride concentration, were examined. Both AIP and CIP delivery of the drug was enhanced compared to passive permeation. Importantly, the INS transdermal transfer during the CIP experiment was significantly increased compared to that of AIP stimulation due to the repulsion of negatively charged INS and the cathode, as well as the nature of PPy. The INS-loaded WS-PPyNPs under both stimuli permeated more than 750 µg cm^−2^ INS on average for 48 h. Moreover, the WS-PPyNPs exhibit good P_app_ value for INS delivery (1.25 × 10^−5^ cm s^−1^) using CIP.

## Figures and Tables

**Table 1:** Skin permeation parameters for INS after 48 h; Average Values ± SD, *n* = 3.

Experiment	Q48h (µg cm^−2^)	INS Flux (µg cm^−2^ h^−1^)	Lag Time (min)	P_app_ (cm s^−1^)	EF
Passive	17.87 ± 4.69	0.36 ± 0.09	68 ± 12	2.88 (± 0.79) × 10^−7^	−
AIP	520.0 ± 136.4	9.59 ± 2.65	91 ± 13	7.68 (± 2.15) × 10^−6^	26.7 ± 10.9
CIP	834.6 ± 218.9	15.67 ± 4.24	105 ± 18	1.25 (± 0.37) × 10^−5^	43.4 ± 18.3

## Supplementary Materials

The following are available online at https://www.mdpi.com/article/10.3390/ijms222212479/s1.
