# Supplementary material for: Controlled Transdermal Iontophoresis of Insulin from Water-Soluble Polypyrrole Nanoparticles: An In Vitro Study
Source: Int J Mol Sci. 2021 Nov 19;22(22):12479. doi: 10.3390/ijms222212479 (PMC8621898; doi:10.3390/ijms222212479)
Supplement: Supplementary file 1 [file ijms-22-12479-s001.zip › ijms-1375803-supplementary.pdf]

Supporting information:

Controlled Transdermal Iontophoresis of Insulin from Water-Soluble Polypyrrole Nanoparticles: An In-Vitro Study

Kamran Tari [1,a], Soroush Khamoushian [2,a], Tayyebeh Madrakian [*,2,3], Abbas Afkhami [2,4], Marek J. Łos [*,3,5,] Arash Ghoorchian [2], Mohammad Reza Samarghandi [*,1] and Saeid Ghavami [6]

1 Department of Environmental Health Engineering, Faculty of Health and Research Center for Health Sciences, Hamadan University of Medical Sciences, Hamadan, Iran; (KT) kamerantari@yahoo.com , (MRS) samarghandi@umsha.ac.ir

2 Faculty of Chemistry, Bu-Ali Sina University, Hamedan, Iran; (SH) soroushkhamoushyian24@gmail.com , (AG) arash_ghoorchian@yahoo.com

3 Autophagy Research Center, Shiraz University of Medical Sciences, Shiraz, Iran; (TM) madrakian@basu.ac.ir

4 D-8 International University, Hamedan, Iran; (AA) afkhami@basu.ac.ir

5 Biotechnology Center, 8 Krzywousty St., Silesian University of Technology, Gliwice, Poland; (MJL) mjelos@gmail.com

6 Research Institute of Oncology and Hematology, Cancer Care Manitoba, University of Manitoba, Winnipeg, Canada; (SG) saeid.ghavami@gmail.com

a These authors share equal first authorship,

* Correspondence: (TM) madrakian@basu.ac.ir , (MRS) samarghandi@umsha.ac.ir , (MJL) mjelos@gmail.com

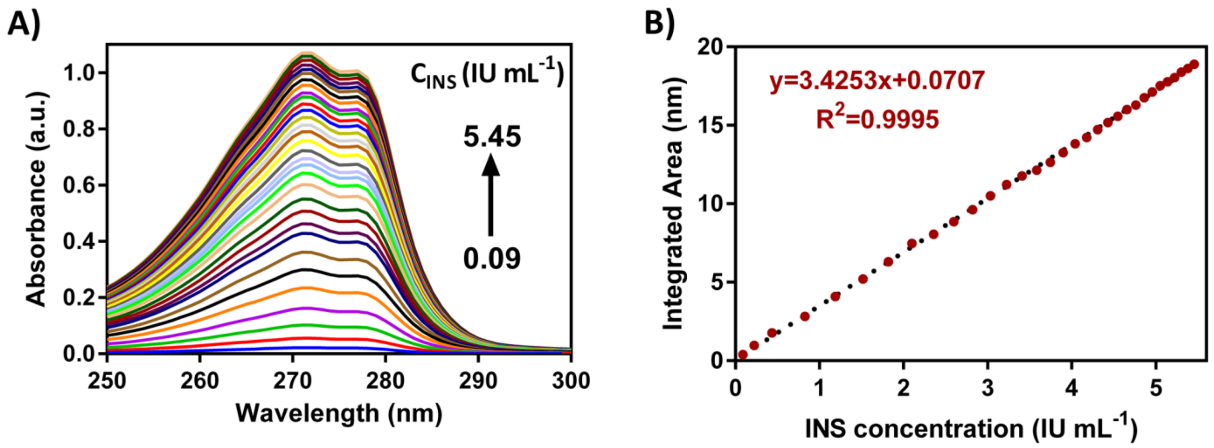

Figure S1. (**A**) The absorbance spectra of PBS (pH 7.4) in the presence of various concentrations of INS. (**B**) The corresponding relationships between the integrated area and concentrations of INS.

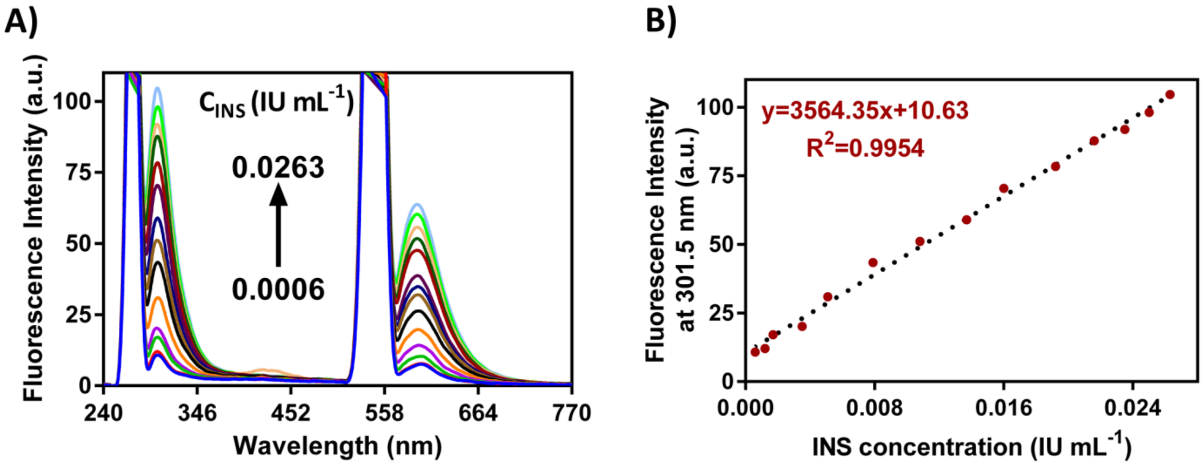

Figure S2. (**A**) The fluorescence spectra of PBS (pH 7.4) in the presence of various concentrations of INS. (**B**) The corresponding relationships between the fluorescence intensity at 301.5 nm and concentrations of INS.

Table S1. EE % values obtained using fluorescence spectroscopy.

INS concentration (IU mL^{-1})	EE (%)
1.0	ND[a]
10	92.41
50	83.63
70	62.46

[a] ND: not detectable.
